# Winds of change a tale of: asthma and microbiome

**DOI:** 10.3389/fmicb.2023.1295215

**Published:** 2023-12-11

**Authors:** David Galeana-Cadena, Itzel Alejandra Gómez-García, Karen Gabriel Lopez-Salinas, Valeria Irineo-Moreno, Fabiola Jiménez-Juárez, Alan Rodrigo Tapia-García, Carlos Alberto Boyzo-Cortes, Melvin Barish Matías-Martínez, Luis Jiménez-Alvarez, Joaquín Zúñiga, Angel Camarena

**Affiliations:** ^1^Laboratorio de Inmunobiología y Genética, Instituto Nacional de Enfermedades Respiratorias Ismael Cosío Villegas (INER), Mexico City, Mexico; ^2^Tecnologico de Monterrey, Escuela de Medicina y Ciencias de la Salud, Mexico City, Mexico; ^3^Red de Medicina para la Educación, el Desarrollo y la Investigación Científica de Iztacala, Facultad de Estudios Superiores Iztacala, Universidad Nacional Autónoma de México, Mexico City, Mexico

**Keywords:** asthma, microbiota, exacerbations, gut-lung axis, diversity, environmental factors, asthma phenotypes

## Abstract

The role of the microbiome in asthma is highlighted, considering its influence on immune responses and its connection to alterations in asthmatic patients. In this context, we review the variables influencing asthma phenotypes from a microbiome perspective and provide insights into the microbiome’s role in asthma pathogenesis. Previous cohort studies in patients with asthma have shown that the presence of genera such as *Bifidobacterium, Lactobacillus, Faecalibacterium*, and *Bacteroides* in the gut microbiome has been associated with protection against the disease. While, the presence of other genera such as *Haemophilus, Streptococcus, Staphylococcus*, and *Moraxella* in the respiratory microbiome has been implicated in asthma pathogenesis, indicating a potential link between microbial dysbiosis and the development of asthma. Furthermore, respiratory infections have been demonstrated to impact the composition of the upper respiratory tract microbiota, increasing susceptibility to bacterial diseases and potentially triggering asthma exacerbations. By understanding the interplay between the microbiome and asthma, valuable insights into disease mechanisms can be gained, potentially leading to the development of novel therapeutic approaches.

## 1 Introduction

Asthma is a common respiratory disease that affects individuals of all ages. It is now recognized as a condition with several phenotypes and as a group of several distinct diseases, known as endotypes. Some asthma phenotypes that have been described include young individuals with allergies, overweight middle-aged individuals, and elderly individuals with unhealthy aging, among many others. However, their similarities give rise to a common syndrome characterized by reversible airway obstruction, nonspecific airway hyperresponsiveness, and chronic airway inflammation (Kuruvilla et al., 2019; Hizawa, 2023).

The underlying pathogenesis of asthma is extremely complex and diverse, with a significant economic impact due to the need for long-term treatment (Nurmagambetov et al., 2018) and a potential decrease in quality of life. Clinically, asthma is a chronic airways disease characterized by recurrent episodes of wheezing, coughing, thoracic oppression, and dyspnea (GINA Report, 2022). The immune system plays a central role in the pathophysiology of asthma, involving the inflammatory response and sensitivity to allergens (Bush, 2019). Furthermore, recent research has highlighted the importance of the microbiome in the development of the immune response, as it is educated and modified by microorganisms and metabolites of the microbiome (Zheng et al., 2020). On the other hand, respiratory diseases have been associated with decreased microbial diversity, termed dysbiosis, defined as deviation from a normal microbial composition, is associated with a number of adverse biological phenomena, sometimes with clinical consequences (Natalini et al., 2023). Respiratory and gut dysbiosis modifies immune system responses which influences inflammation in the lungs, leading to a potential role in asthma pathophysiology, phenotypes, and clinical outcomes (Ver Heul et al., 2019; Hufnagl et al., 2020). Typically, attention is usually focused on a single point, involving the analysis of microbiota from singular anatomical sites during specific developmental stages or, in certain instances, restricting the focus solely to pediatric and adult cohorts (Zimmermann et al., 2019; Losol et al., 2021; Aldriwesh et al., 2023). However, a noteworthy challenge arises when endeavoring to amalgamate shared findings from diverse studies. While certain commonalities have been identified, their respective implications vary depending upon the contextual framework (Barcik et al., 2020; Lupu et al., 2023; Zhao et al., 2023). Consequently, it has proven to be quite formidable to identify a specific taxonomic group that consistently influences or mitigates the risk factors or clinical presentations of asthma across all scenarios. Thus, we assert the significance of exploring the role of the microbiome within the context of its development, eschewing the presumption that a particular taxonomic group universally assumes an identical role in all circumstances. In this context, we have conducted a comprehensive review to explore the diverse roles of the microbiome in relation to the phenotypes and endotypes of asthma throughout human growth and development, encompassing prenatal factors, birth, childhood, adolescence, adulthood, and the elderly.

## 2 Microbiome

The microbiome encompasses the microbiota, their genetic material, metabolites, and the surrounding microenvironment (Berg et al., 2020). Each body site has its own distinct composition and complexity of microorganisms. When evaluating the microbiome based on sequencing data, two important terms are often employed: alpha diversity, which measures the number and abundance of microorganisms in a specific sample or site, and beta diversity, which quantifies the variation in microorganisms between different samples or sites (Finotello et al., 2018). Within the microbiome, a multitude of commensal microorganisms have undergone co-evolution with human cells; giving rise to complex, dynamic, interdependent, and context-dependent relationships essential for maintaining ecosystem balance within their respective communities, a state referred to as eubiosis (Iebba et al., 2016). Eubiosis and host-microbiome relationship influence various host functions, including metabolism, immunity, circadian rhythms, nutritional responses, and homeostasis (Zheng et al., 2020). In order to achieve its functions, human cells engage in intricate communication mechanisms through a system-system axis, enabling coordinated responses across different organs such as the gut-brain axis and gut-lung axis (Suganya and Koo, 2020; Ahlawat et al., 2021).

**Figure 1:**
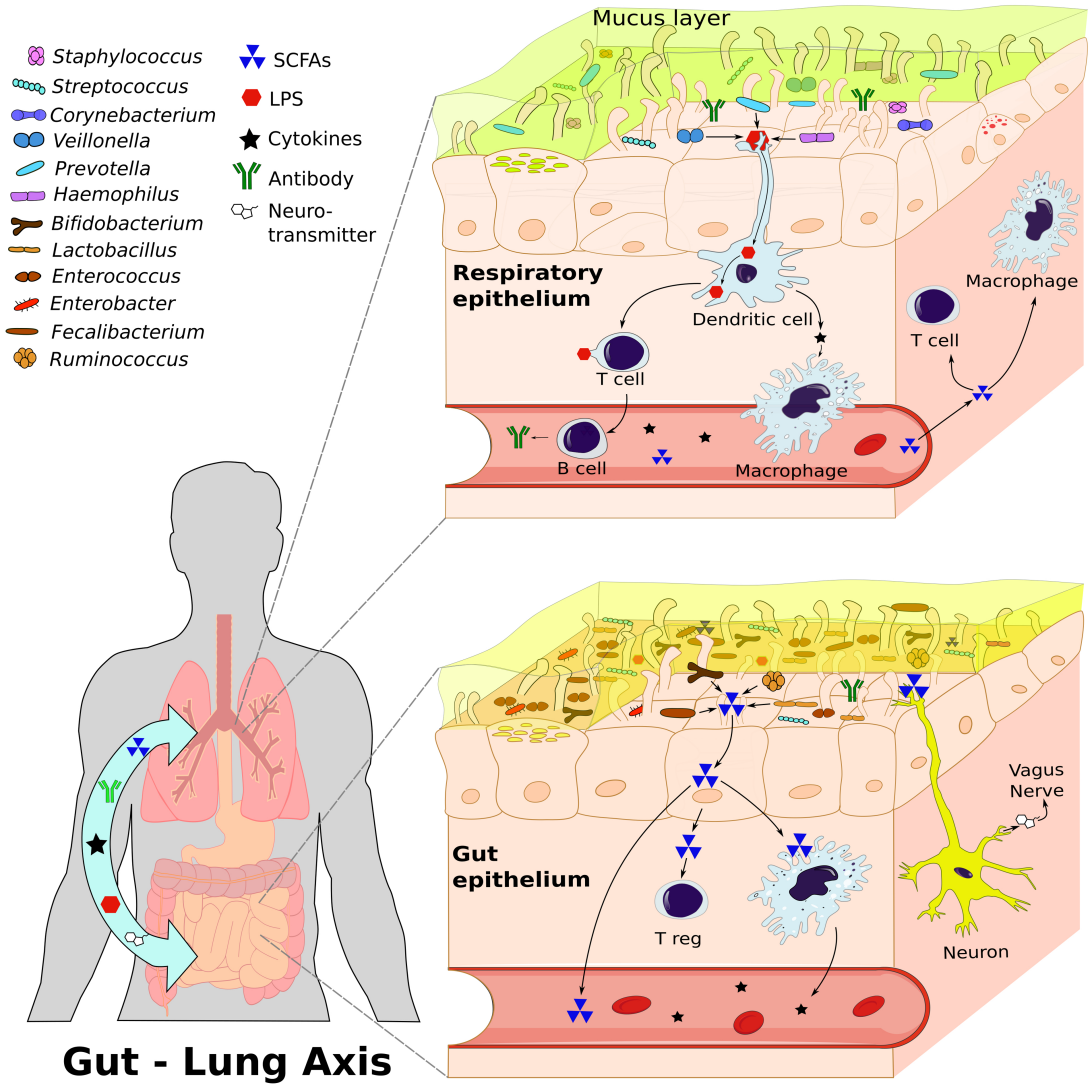
The Gut-Lung Axis: A bidirectional communication network between the gastrointestinal tract and the respiratory system involving interactions among immune cells, neurons, microbiota, and signaling molecules. This figure illustrates the composition of both lung and gut microbiota and the dynamic interactions within this axis; highlighting the role of microbiome dependent communication. This communication stimulates the immune cells through signal molecules, such as short-chain fatty acids (SCFAs), lipopolysaccharide (LPS), and cytokines. Additionally, through the vagus nerve, the microbiome stimulates the production of neurotransmitters. All these molecules can travel through the bloodstream to influence both the gut and lung systems. Created with inkscape.

**Figure 2:**
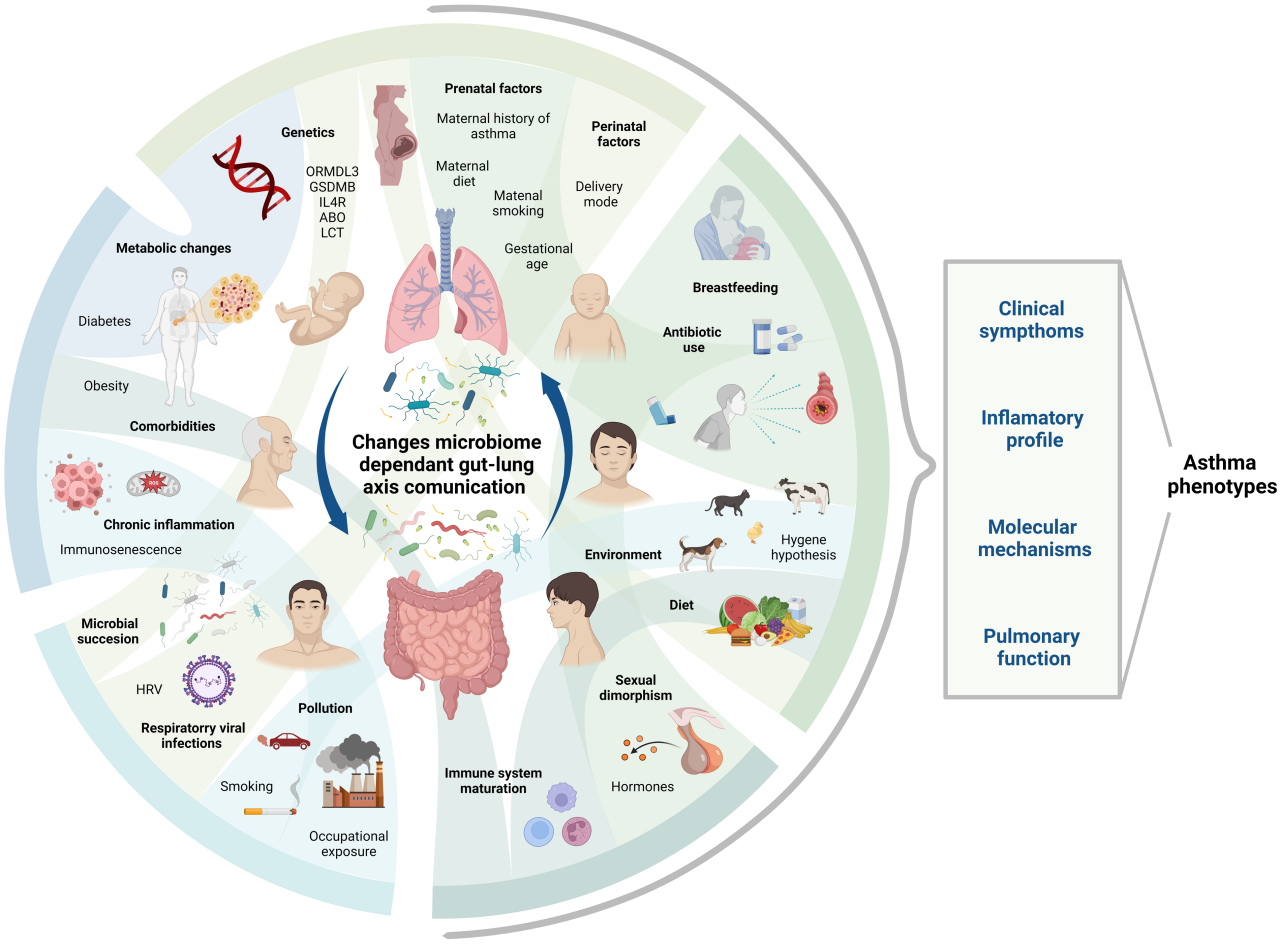
Asthma phenotype and factors determining microbiome through life stages. This figure shows some of the factors influencing asthma phenotype and microbiome composition during different life stages. Their complex interactions require study in order to comprehend their role in the context of gut-lung axis communication via microbiome and asthma development. Created with BioRender.com. Bio render agreement number: PT264MFOUQ.

The gut-lung axis plays a significant role in respiratory pathologies as it establishes a bidirectional pathway for the transmission of internal and external factors, creating a signaling network that can influence systemic functions and responses (Enaud et al., 2020). The composition of the microbiome on mucosal surfaces, including the gastrointestinal and respiratory tracts, is highly dynamic. The gut microbiota consists of approximately 3,594 species, primarily classified under the phyla *Actinobacteria, Bacteroidota, Firmicutes*, and *Proteobacteria* (Leviatan et al., 2022). Contrary to previous beliefs, it is now known that the human lung harbors a distinct lung microbiota, mainly composed of genera such as *Prevotella, Veillonella*, and *Streptococcus*, thanks to advancements in sequencing techniques (Dickson et al., 2016; Yagi et al., 2021; Natalini et al., 2023). Molecular signals, including short-chain fatty acids (SCFAs) produced by *Bifidobacterium, Lactobacillus, Faecalibacterium*, and *Ruminococcus* (Tsukuda et al., 2021), facilitate communication between these organs through circulation or via the vagus nerve. Several species have also shown marked effects as neuromodulators and neurotransmitters such as monoamines, serotonin, and brain-derived neurotrophic factor (Suganya and Koo, 2020). *Lactobacillus* is strongly involved in both the gut-lung axis and the brain-gut axis (Rastogi and Singh, 2022), and can produce GABA and activate receptor expression, leading to cognitive enhancement via the vagus nerve (Breit et al., 2018). Oral ingestion of *L. rhamnosus* and *L. murinus* promotes migration of T Regulatory Cell (Treg) to the lungs and blocks the Th2 response (Zhang et al., 2018a; Han et al., 2021), thereby reducing respiratory inflammation (Figure 1). Wang et al. also prove that *L. fermentum* can reduce the expression of Toll-like Receptor 2 and Toll Like Receptor 4 in OVA mice model, with concurrent reduction in inflammatory cell infiltration and alveolar swelling (Wang et al., 2022). The composition and function of the microbiome in both the intestine and the lung are influenced by a variety of factors, including genetics, the immune system, pregnancy, birth conditions, age, dietary habits, pollution, antibiotics, and lifestyle (Martino et al., 2022; Figure 2).

## 3 Microbiome and asthma: the importance of the beginning

The journey of an individual begins with the intricate process of fertilization and genetic recombination, which sets the foundation for the expression of unique characteristics that will define their existence. However, the conditions of birth, growth, and development that an individual experience will play a crucial role in shaping their traits and creating the environment in which they thrive; shaping an individual’s life journey.

### 3.1 Genetics factors

Asthma is considered a chronic complex disease that is the consequence of an interaction between genetic and environmental factors. Over 100 genes have been associated with asthma and the features of the disease; however, there is marked variability in replication attempts in independent studies. Genetic variants on chromosome 17q21 near to ORMDL3/GSDMB locus have been associated with childhood-onset asthma (Ntontsi et al., 2021; Afzal et al., 2023). Kumar et al. highlighted the association between the variant rs1805011 in the IL-4 receptor gene and Th1/Th2 differentiation, which increases susceptibility to asthma (Kumar et al., 2015). This finding shed light on the intricate interplay between genetic factors and T-cell responses.

In this context, there has recently been growing interest in understanding the role of host genetics in shaping the gut microbiome, and several studies have shed light on this complex interplay. Boulund et al., identified significant correspondences in microbial taxa that are partly regulated by host genotype, with host genes associated with these taxa being related to secretion-metabolism, signaling-transport, and immunity (Boulund et al., 2022). Similarly, Lopera-Mayá et al., conducted a genome-wide association study to comprehensively characterize the effects of host genetics on the gut microbiome, discovering two study-wide significant signals near the Lactase and ABO genes with the *Bifidobacterium* and *Collinsella* genera, respectively (Lopera-Maya et al., 2022). Rühlemann et al., reported an association between the *Prevotella* genus in asthma and ABO blood groups (Rühlemann et al., 2021). Additionally, Ahluwalia et al. proposed that variations in the Fucosyltransferase 2 (FUT2) and ABO genes, along with epistatic effects, may contribute to an increased risk of early childhood asthma (Ahluwalia et al., 2020). They suggest that the expression of AB antigens in the respiratory epithelium and *Streptococcus pneumoniae* infection may be involved. Kurilshikov et al., conducted a genome-wide association study investigating human host genetic variation’s impact on microbial taxa. They identified 31 loci that influence the gut microbiome. Among these loci, the LCT gene stands out as being particularly significant for the Bifidobacterium genus (Kurilshikov et al., 2021). Regarding the gut microbiome, host genetics, and asthma, Li et al., described that, through a two-sample Mendelian randomization analysis, they predicted a positive correlation between the gut species *Barnesiella* and *RuminococcaceaeUCG014* genera and the risk of asthma. Furthermore, they found that *Akkermansia* reduced the risk of adult-onset asthma (Li et al., 2023). Perez-Garcia et al., through a study of microbiome- genome wide association (mbGWAS) and microbiome quantitative trait loci (mbQTL) analysis, reported the identification of polymorphisms in the APOBEC3B-APOBEC3c, TRIM24, and TPST2 genes that are associated with asthma comorbidities. These polymorphisms were found to be mbQTLs related to S*treptococcus, Tannerella*, and *Campylobacter* in the upper airway (Perez-Garcia et al., 2020). Nevertheless, as proposed by Chen et al., it is suggested that risk variants on 17q12-21 and perturbations in the maturation of intestinal microbiota associate independently and exhibit additive effects on the risk of asthma development (Chen et al., 2023). Therefore, more studies are needed to define the role of host genetics in microbial diversity and asthma.

### 3.2 Prenatal factors

Prenatal factors have been associated with increased susceptibility to asthma, as demonstrated by cross-sectional studies and systematic reviews (Castro-Rodriguez et al., 2016; Arif and Veri, 2019; Esmeraldino et al., 2022). These factors include maternal smoking (Moradzadeh et al., 2018), maternal history of asthma (García-Serna et al., 2021), antibiotic use during pregnancy (Alhasan et al., 2020), maternal diet (Gray et al., 2017), and maternal stress (Van De Loo et al., 2016; Douros et al., 2017), among others.

Furthermore, the human fetal immune system initiates its development during the first four weeks of gestation (Park et al., 2020). Early exposures to metabolites from the maternal microbiota contribute to establishing a functional immune response at birth (Donald and Finlay, 2023). In a murine model, the transfer of antigen-specific IgG during fetal development provides protection to the offspring against allergic airway inflammation (Nakata et al., 2010). This suggests that antibodies play a dual role by not only safeguarding against particular pathogens but also contributing to the establishment of tolerance and recognition of commensal bacteria that will colonize the newborn (Koch et al., 2016; Macpherson et al., 2017; Mimoun et al., 2020). Additionally, the maternal microbiota prepares the newborn for host-microbial mutualism, which results from microbial metabolite transfer. By transiently colonizing pregnant female mice, with *E. coli* HA107, the maternal microbiota shapes the immune system of the offspring. Maternal microbial metabolites increases intestinal group 3 innate lymphoid cells and F4/80 + CD11c (Gomez de Agüero et al., 2016). Regarding the translocation of maternal microorganisms for fetal colonization, Jimenez et al. designed an experiment using a labeled strain of *Enterococcus faecium*. They orally inoculated pregnant mice and successfully recovered the microorganism from the meconium and amniotic fluid of the cesarean-born animals (Jiménez et al., 2008). However, a systematic review reports that the meconium microbiota in humans begins to develop after birth (Turunen et al., 2023).

On the other hand, the administration of antibiotics closer to parturition has been shown to have a significant impact on the diversity of both neonatal and maternal microbiota. Specifically, it has been found to increase the abundance of the phylum Proteobacteria in neonates exposed to antibiotics. In contrast, unexposed neonates tend to have a dominance of phylum Firmicutes with families such as Streptococcaceae and Lactobacillaceae (Stiemsma and Michels, 2017).

Furthermore, antibiotics can disrupt the maternal microbiome in the vagina. For example, the administration of antibiotics to the mother during the intrapartum period before birth, as well as the duration of rupture of membranes (ROM), have been found to be significantly associated with a decreased transmission rate of *Lactobacillus*-dominant mixed flora to neonates (Keski-Nisula et al., 2013). Considering all of the above, we believe it is necessary to continue investigating prenatal factors in the development of the newborn’s immune system and microbiome.

### 3.3 Perinatal factors

The process of birth represents a complex series of changes, including the first interaction between the newborn and the microbiome along with the onset of immune system training. In the beginning, we were born with an immature immune system mainly dependent on the innate immune system. Neonatal dendritic cells (DCs) exhibit adult levels of the immunoregulatory cytokine IL-10 when stimulated with lipopolysaccharides (LPS) from the maternal microbiome, but they are less proficient in promoting T helper 1 (Th1) cell differentiation due to delayed IL-12 production. Instead, the neonatal immune system tends to favor immunoregulatory and Th2 cell responses. This serves as a protective mechanism to prevent excessive inflammatory responses to novel antigens found in the environment and commensal microorganisms of their own microbiota (Donald and Finlay, 2023).

Hygiene hypothesis proposes that during early life, exposure to exogenous determinants such as breastfeeding, environment and microbiome plays a protective role against allergic diseases by facilitating the maturation of the immune system. This critical period of exposure spans from perinatal life until school-age (Liu, 2007; Garn et al., 2021; Pfefferle et al., 2021). In essence, it suggests that a lack of exposure to microorganisms may result in impaired immune tolerance development.

The initial colonization of microorganisms is closely linked to the mode of delivery and gestational age at birth (Castro-Rodriguez et al., 2016). When a baby is born vaginally, they are immediately exposed to microorganisms primarily inhabiting the maternal gut lumen and vagina, such as *Lactobacillus, Bacteroides*, and *Bifidobacterium* genera (Kalbermatter et al., 2021). Consequently, the gut microbiota of newborns born vaginally tends to be similar to that of their mothers (Song et al., 2021; Yao et al., 2021). Within the first days after birth, Clostridiaceae, Enterococcaceae, and Streptococcaceae families are observed, followed by the appearance of *Bacteroides* and *Bifidobacterium* in the guts of 40% of infants after the third day (Yao et al., 2021). In contrast, the colonization of the upper respiratory tract initiates with *Staphylococcus* and *Streptococcus* followed by the proliferation of *Moraxella, Corynebacterium, Dolosigranulum*, and/or *Haemophilus* species (Bosch et al., 2016), which are associated with reduced risk of respiratory symptoms (Biesbroek et al., 2014; Teo et al., 2015).

However, the colonization process can be interrupted for several reasons, mainly Cesarean section (C-section) delivery. Compared to babies born vaginally, infants delivered via C-section share roughly 30% fewer bacterial species with their mothers (Kalbermatter et al., 2021). In the upper respiratory tract Bosh et al., reported a lower abundance of *Corynebacterium and Dolosigranulum,* especially in the first months of life (Bosch et al., 2016). In gut microbiota, these newborns primarily harbor microbes from the maternal skin and antibiotic-resistant bacteria from the hospital environment, including *Staphylococcus, Streptococcus*, and *Clostridium*, which can alter its maturation (Rutayisire et al., 2016).

In fact, evidence suggests that alteration of microbiota by C-section, especially the elective one, is associated with alterations in the immune system, increasing the risk for developing asthma, allergies, type I diabetes mellitus, and celiac disease (Salas Garcia et al., 2018; Ferllini Montealegre et al., 2019; Kumbhare et al., 2019). Słabuszewska-Jóźwiak et al. in a meta-analysis study for C-section delivery and asthma in offspring, reported an odds ratio of 1.23 (95%CI 1.14–1.33, *p* < 0.00001) and a higher frequency of asthma in the C-section delivered children (Słabuszewska-Jóźwiak et al., 2020). Another cohort study revealed a relative risk of 1.11 (95% CI 1.00 to 1.25) in children with partially controlled asthma which increased to 1.8 (95% CI 1.00 to 1.39) in children with uncontrolled asthma (Moore et al., 2023). This risk could be explained because children who maintain a microbiota associated with C-section display a different immune response during respiratory symptom episodes, with lower levels of TNF-a, IL-4, IL-13, or IL-1b. Additionally, infants delivered by C-section to long-term have high levels of IgE making them susceptible to asthma or the development of allergies (Stokholm et al., 2018).

C-section delivery is more common in premature babies (infants born before 37 weeks of gestation) and has been associated with the development of various health problems, including asthma. Also, preterm can lead to delayed and reduced gut colonization by beneficial bacteria like *Bacteroides, Bifidobacterium*, and *Lactobacillus* species. This creates an opportunity for other bacteria, such as family Clostridiaceae, to colonize gut lumen, potentially contributing to the development of asthma and allergic disorders (Zimmermann et al., 2019). Zhang et al., through a meta-analysis reported that prematures have up to a 36% higher risk of asthma compared to infants born at term estimated (Zhang et al., 2018b). Furthermore, formula feeding in premature babies reduces overall gut microbial diversity and reduces *Bifidobacterium* levels (Healy et al., 2022). A Swiss cohort study that followed 4, millions of births up to 46 years of age, described that the risk of developing asthma increases as the age of the infant at birth decreases, with a probability 1.5 to 2.5 times greater than that of a full-term newborn (Crump et al., 2023). Moreover, premature infants often experience reduced lung function and structural alterations in the lungs, leading to airflow problems (Arroyas et al., 2020).

Furthermore, preterm infants exhibit elevated rates of hospitalization related to Respiratory Syncytial Virus (RSV), admissions to the intensive care unit, the need for mechanical ventilation, and extended hospital stays when contrasted with full-term infants (Anderson et al., 2017). In a multicenter prospective cohort study involving 221 infants affected by RSV bronchiolitis, Raita et al. identified an endotype characterized by several distinctive features. This endotype included a high prevalence of parental asthma, IgE sensitization, and concurrent rhinovirus (HRV) infection. Notably, the co-dominance of *Streptococcus pneumoniae* and *Moraxella catarrhalis* in the nasopharynx, along with an elevated IFN-α and -γ response, were also prominent characteristics. This particular endotype was significantly associated with an increased risk of developing childhood asthma. It’s worth noting that among these patients, 22.2% were born prematurely (Raita et al., 2021). Importantly, infection with HRV-C has been linked to more severe asthma exacerbations compared to HRV-A and HRV-B (Bizzintino et al., 2011).

These infections reduce Th1 and IFN-γ responses, leading to an increase in Th2 responses, which promote inflammation and bronchoconstriction (Pinto et al., 2006). Additionally, premature infants often have elevated levels of IL-17 from Th17 cells, which further exacerbates inflammation, resulting in characteristics of both eosinophilic asthma (IL-4 and IL-13) and neutrophilic asthma (IL-17), leading to a combined type of asthma (Chesné et al., 2014; Anderson et al., 2023).

While progress has been made in the study of the aforementioned factors, additional long-term longitudinal studies are still needed to understand the effects of changes in the microbiome during the perinatal period.

## 4 Infancy and childhood, imprint development

Childhood asthma is a prevalent and complex respiratory condition that affects millions of children worldwide, posing significant health challenges and burdens. According to literature review, early-life microbiota disturbances can lead to immune alterations, including T-reg cells proliferation, Th17 response, and IgE response in humans (Lee and Kim, 2017). Furthermore, the gut and airway microbiota in the first year of life has been reported to induce T-reg cells that enhance tolerogenic immunity in infants at high risk for asthma (Busse and Rosenwasser, 2003). Multiple investigations have shown differences between the lung and gut microbiome of individuals with established asthma vs. healthy subjects, being the population with asthma the ones with lower bacterial diversity (Marsland et al., 2015; Carr et al., 2019; Liu et al., 2020). Case–control studies have associated gut dysbiosis with a reduction in the specific genera *Faecalibacterium, Bifidobacterium, Lachnospira, Veillonella*, and *Rothia* (Yap et al., 2014; Arrieta et al., 2015). Therefore, systematic review highlights the importance of establishing a healthy microbiota during the first years of life. Notably, breastfeeding (Doherty et al., 2018) and supplementation with *Lactobacillus* (Durack et al., 2018; Alliet et al., 2022), have long-lasting potential for immune development and reduce the risk of developing asthma and allergic conditions.

### 4.1 Breastfeeding and changes in diet

Evidence suggests that the microbiome in children is influenced to a greater extent by first feeding method and dietary intake. Human milk, contains immunomodulators and anti-inflammatory agents such as alpha-tocopherol, beta-casomorphins, prolactin, lactoferrin, lysozyme, antioxidants, cytokines, and secretory IgA (Miliku and Azad, 2018), which promote the proper development of both mucosal and systemic immune systems, playing an important role in shaping a more robust immune system compared to the immune system of formula fed infants (Munblit et al., 2017; Domenici and Vierucci, 2022).

The presence of certain bacterial genera, including S*taphylococcus, Streptococcus, Lactobacillus,* and *Bifidobacterium*, has been consistently reported in human milk (Boquien, 2018; Lyons et al., 2020). According to a randomized double-blind trial in Sweden, the richness of bacterial species in breast milk appears to be critical in preventing the overgrowth of potentially harmful species associated with asthma development like *Enterococcus* (Dzidic et al., 2020). Moreover, *Bifidobacterium*, a common genus in breast milk, has demonstrated anti-inflammatory properties by promoting the production of anti-inflammatory cytokines, inhibiting Th2 immune responses, and suppressing IgE production, which further supports the notion that breast milk contributes to reducing asthma risk (Eslami et al., 2020). In contrast, a cohort made in Korea shows that infants who are formula-fed demonstrate early diversification of their gut microbiota, accompanied by decreased levels of *Bifidobacteria* and increased abundance of *Escherichia, Veillonella*, and *Enterococcus* (Lee et al., 2015). Compared with direct breastfeeding, any other mode of infant feeding (including formula) was associated with a higher occurrence of opportunistic pathogens, antibiotic resistance, and an increased risk of asthma (Klopp et al., 2017; Pärnänen et al., 2022).

While all these suggest breastfeeding is protective against asthma in children (Ahmadizar et al., 2017), others have found no significant association at all (Elliott et al., 2009). These discrepancies may be attributed to several factors, including differences in study design and confounding variables such as breast milk composition, which can vary across populations, potentially explaining the observed variations in breastfeeding effects of breastfeeding across studies (Miliku and Azad, 2018). It is important to continue research efforts to better understand the complex relationship between breastfeeding and asthma, taking into account microbiome immune training.

An important event during infancy that impacts the development of the gut microbiome is the introduction of solid food and the cessation of breastfeeding. This shift results in an increase in Lachnospiraceae and Ruminococcaceae, while causing a decrease in Bifidobacterium, Enterobacteriaceae, Enterococcaceae, Lactobacillaceae, Veillonellaceae, Clostridiaceae, and an overall increase in microbial diversity (Laursen et al., 2017). This transition is both necessary and advantageous. It fosters the development of a microbial community better suited to extract energy and process a diet that is no longer reliant on milk, transitioning to a diet rich in fiber and protein (Dong and Gupta, 2019).

Dietary intake has been shown to influence systemic inflammation. The Western diet is characterized by a lack of antioxidants and high levels of fatty acids. This diet has been associated with the promotion of oxidative stress and the activation of inflammatory cascades through receptors such as Toll-like receptor 4 (TLR4), leading to a pro-inflammatory environment, according to a cohort study which compares asthmatic patients vs. healthy controls (Wood et al., 2015). Additionally, the consumption of high-fat mixed meals has been found to increase sputum neutrophils in patients with asthma, particularly observed 4 h after the meal (Wood et al., 2011). Schroeder et al. conducted a study using a mouse model fed a Western diet, which resulted in a reduction in the populations of *Bifidobacterium, Sutturella*, and *Akkermansia* genus. Simultaneously, there was an increase in the Clostridiales order and the *Lactobacillus* and *Oscillospira* genus. Notably, the decrease in *Bifidobacterium* taxa coincided with the onset of mucus defects in this model (Schroeder et al., 2018). Moreover, a Western diet can lead to endotoxemia, contributing to intestinal barrier impairment and increased levels of bacterial lipopolysaccharides (LPS), consequently leading to heightened inflammatory signaling (Pendyala et al., 2012).

Conversely, the Mediterranean diet characterized by a diverse range of fruits, olive oil, vegetables, and whole grain cereals is believed to create an anti-inflammatory environment. This is attributed to the presence of dietary fiber, unsaturated fatty acids, such as monounsaturated fatty acids and Omega-3, as well as the abundance of antioxidants. In a meta-analysis conducted by Garcia-Marcos et al., it was found that the Mediterranean diet was significantly associated with a reduced prevalence of asthma (OR: 0.86; 95% CI 0.78–0.95; *p* = 0.004) (Garcia-Marcos et al., 2013). Similarly, a meta-analysis by Zhang et al., reported protective effects of a Mediterranean diet on asthma (OR = 0.88; 95% CI: 0.79–0.97; *p* = 0.014) (Zhang et al., 2023). However, further research is required to gain a deeper understanding of the specific role of the microbiome in individuals with asthma who adhere to a Mediterranean diet. Moreover, high-fiber diets have been associated with an increase in the colonic Bacteroidetes and Actinobacteria phyla, while the Firmicutes and Proteobacteria phyla decreased. These shifts in microbial composition provide protection against allergic responses, as demonstrated in mouse models (Zhang et al., 2016). Dietary fibers, such as pectin, are fermented by commensal gut bacteria, which produce metabolites including SCFAs, which mediate anti-inflammatory responses (Blanco-Pérez et al., 2021). Ketogenic diet during pregnancy, lactation, and early childhood have been related to low risk of developing asthma due to changes in epigenetic markers. For instance, Bifidobacterium and Lactobacillus species produce SCFAs, which exert anti-inflammatory effects on immune cells through an epigenetic mechanism that involves the inhibition of histone deacetylases associated with butyrate. This modulation of the immune response leads to a reduced risk of asthma (Alsharairi, 2020).

### 4.2 Environment influences on children’s development and growth

Numerous epidemiological studies have shown that living on a farm during early childhood is associated with a reduced risk of developing asthma in childhood (Stein et al., 2016; House et al., 2017; Depner et al., 2020). Under these circumstances, Bifidobacterium and Lactobacillus species produce SCFAs, which exert anti-inflammatory effects on immune cells through an epigenetic mechanism involving the inhibition of histone deacetylases associated with butyrate. In particular, a study that compares the Amish and Hutterites populations, who had similar genetic backgrounds but different farming practices, found that the Amish, who followed traditional farming practices involving high microbial exposures to animals, had a lower risk of childhood asthma. In contrast, the Hutterites, who practiced industrialized farming, did not show the same protective effect (Stein et al., 2016).

A study conducted across 14 countries compared the effects of farm environments and inner city environments on the development of allergic diseases (Campbell et al., 2017). The findings revealed that exposure to a farm environment was associated with a protective effect against allergic diseases. One of the key factors identified by other authors in this protective effect was the consumption of farm milk, which has been reported to contain higher levels of gram-negative bacteria such as *Escherichia coli, Pseudomonas,* and *Klebsiella*, as well as lipopolysaccharides (LPS) (Garedew et al., 2012; Gehring et al., 2020). Endotoxins as LPS, most commonly studied in combination with dust, induce a Th2 response in mice and exacerbate lung eosinophilia via TLR4 pathways, which can result protective against allergic diseases, such as asthma (Ren et al., 2019). A low dose but continuous exposure to an endotoxin, was protective in a mouse model of asthma. This suggests that by the time children reach school age, they will exhibit a marked suppression of the capacity of a Th2 response as a consequence of long-term exposure to environmental endotoxins (Braun-Fahrländer et al., 2002).

Furthermore, it has been considered that growing up with pets and siblings exerts complex changes in microbiota composition, a longitudinal study comparing fecal microbiota composition in infants reports that subjects exposed to both pets and siblings tended to have low relative abundance of family Bifidobacteriaceae and other bacterias (Azad et al., 2013). These changes in gut microbiota result in the development of a healthy immune system that can end up being protective against asthma and allergy development. Siblings are another of the most important determinants in the development of microbiota during early childhood, due to its impact on alpha and beta diversity (Christensen et al., 2022). However, there is also a risk of exacerbating asthma if the child has a pet-specific allergic sensitization (Pinot de Moira et al., 2022). Moreover, at least 56 bacterial genera were significantly more abundant in homes with dogs, such as *Prevotella, Porphyromonas, Moraxella,* and *Bacteroides*, which were found in the mouth and feces of the animals and also in their owners (Barberán et al., 2015; Hufnagl et al., 2020).

The relationship between hygiene hypothesis and its effect at immunology level can be explained by different mechanisms. First, exposure to a larger diversity of bacterial species enables the development of a balanced immune response. Second, these exposures might serve as the starting point for the development of the infant gastrointestinal microbiome. Third, dietary transitions that facilitate immune tolerance to food nutrients. Fourth, the environment in which one grows. Lack of appropriate microbial and diet exposure might be related with allergic diseases such as asthma (Penders et al., 2006; Fujimura et al., 2010; Garn et al., 2021; Pfefferle et al., 2021).

### 4.3 Antibiotics: use and abuse

Nowadays, antibiotics play an important role in modern medicine and have improved the prognosis of an endless number of patients, however, we must not forget they have several adverse effects to take into consideration, such as antibiotic resistance and microbiome alteration (Ni et al., 2019).

Several studies have shown that the use of antibiotics in early childhood increases the risk for developing asthma up to 2.3 times (Kim et al., 2018; Lin et al., 2020; Li et al., 2023), these risks increase even more with the number of antibiotics courses prescribed. In infants (<1 year) antibiotic use is associated with a 24% higher incidence of asthma for every 10% increase in prescriptions (Patrick et al., 2020). Among the most prescribed are B-lactams such as amoxicillin (2-fold), followed by macrolides, second-generation cephalosporins (2.7 fold) and sulfonamides (Marra et al., 2009; Li et al., 2023).

A cohort of 697 children demonstrates that the exposure to 2 or more antibiotics from B-lactam group, during the first year of life was related with an increment in the risk of asthma together with longitudinal changes in the nasal microbiome, being the most significant change *Moraxella* sparsity (Toivonen et al., 2021). In another experimental study where mice were exposed to antibiotic treatment there was an increase in Phylum Proteobacteria, and a decrease in Bacteroidetes and Firmicutes, in the same model, 2 weeks after antibiotic-free period Firmicutes and Bacteroidetes returned to dominance, however Proteobacteria turned up to be relatively increased, compared to controls (Antonopoulos et al., 2009), change that has been found in fecal samples from both allergic and non-allergic asthmatic subjects compared to healthy ones (Zheng et al., 2022).

Specifically, regarding amoxicillin antibiotic, a clinical trial in humans demonstrates that Clostridia and Firmicutes part of gut microbiome after cessation of treatment with this antibiotic were decreased in abundance. In fact, the use of amoxicillin for long periods of time was related with significant depletion of SCFAs bacterial species even after antibiotic therapy completion (Dhariwal et al., 2023). As mentioned before SCFAs mediate anti-inflammatory responses, explaining their protective effects against asthma and other allergic diseases (Blanco-Pérez et al., 2021).

An experimental study using mouse models demonstrated that the disturbance of the microbiota following antibiotic exposure leads to an elevated presence of fungal microbiota, particularly *Candida albicans*. These fungi are known to produce molecules with immunomodulatory functions, such as prostaglandin-like oxilipin protein (Noverr et al., 2004). Furthermore, the same study reported that the disruption of the microbiota resulted in an upregulation of the Th2 immune response to spores and ovalbumin, indicating a consistent allergic reaction (Noverr et al., 2004).

The complex interplay between antibiotics, microbiome changes, and the development of asthma calls for further research. However, it is true that antibiotics are essential for treating bacterial infections and that due to their impact on human microbiome and the risk they represent for the development of asthma and other allergic diseases their excessive or inappropriate use must be approached with caution, especially in children.

## 5 Adolescence, why does everything change?

Changes are a natural process in the course of life. Adolescence is an important transitioning phase of maturation from childhood to adulthood, comprising the 10th to 19th years according to the WHO. Adolescents experience rapid metabolic, immune, sexual, and psychosocial changes among many others. Adolescent development significantly influences the shift in the prevalence of diseases from childhood to adulthood, including asthma.

### 5.1 Adolescence, sexual dimorphism, and hormones

In puberty, significant changes occur in the metabolic, immune, and hormonal responses, which have lasting effects into adulthood. This crucial period shapes the pattern of immune reactions and hormonal regulation, setting the foundation for future responses. Analyses of gene expression and epigenetic modifications have revealed interactions between genotype and puberty on the expression of B cell (IGKV1-27 in males) and T cell (TRBV30 in females) antigen-recognition proteins, with the influence of genetic factors on gene expression to tend to diminish as puberty progresses. Genes associated with pulmonary function exhibit an upregulation, suggesting potential improvements in respiratory capacity during this stage. However, in females, changes in gene expression related to puberty demonstrate a positive correlation with asthma symptoms and an inverse correlation with pulmonary function. With a notable shift in the immune response from a predominantly innate to a more adaptive pattern in females (Resztak et al., 2023).

Hormones are involved in growth and development, especially during adolescence. Sex hormones, in turn, play a key role in the maturation of many tissues. Male and female endocrine patterns differ, resulting in what is known as sexual dimorphism, which has been studied in multiple etiologies. Recently, many research groups have investigated how this phenomenon affects the immune system and its relation with the microbiome (Ucciferri and Dunn, 2022).

The sex-specific prevalence of asthma changes throughout life. Boys have a higher prevalence of asthma than girls (CDC and Prevention. Asthma data, statistics, and Surveillance, 2020) and are twice as likely to be hospitalized for an asthma exacerbation (Kynyk et al., 2011). This pattern can be explained in part by the fact that they have increased allergic inflammation, elevated serum IgE levels (Borish et al., 2005), and dysanapsis, described as a reduction in airway diameter relative to lung volume (Pagtakhan et al., 1984).

In teenagers (around 11 to 16 years), asthma prevalence decreases in males but increases in females (Genuneit, 2014). It has also been observed that adult women are three times more likely to be hospitalized for an exacerbation than men and that this difference decreases after menopause (Troisi et al., 1995). Fu et al., studied this phenomenon and associated symptom progression in girls with the onset of puberty, at the time when it decreases in boys (Fu et al., 2014). During adolescence hormonal differences between males and females modify physiological aspects as well as abiotic (pH, oxygen levels, nutrition) and biotic factors (immune surveillance, signaling molecules), creating numerous niches that allow for the appearance of microbiome differences with further implications for the host. The best-studied example to date is the intestinal microbiome, which has been strongly implicated in numerous sex-specific physiological processes and diseases (Fuseini and Newcomb, 2017). Also, many studies have shown that alpha diversity tends to be higher in adult females than males, with less in older adults (de la Cuesta-Zuluaga et al., 2019; Takagi et al., 2019). Furthermore, studies have provided evidence supporting the presence of sexual dimorphism in the adult gut microbiome (Shin et al., 2019). In a mouse model, it was observed that androgens had a significant impact on the modulation of the gut microbiome and the glutamine/glutamate ratio (Gao et al., 2021). A study conducted on human dizygotic twins revealed that while male and female infant twins displayed conserved beta diversity of the gut microbiome, differentiation between the sexes became more apparent during puberty (Yatsunenko et al., 2012). Additionally, the beta diversity of the gut microbiomes in pubertal males and females became increasingly similar to the adult microbiomes of their respective sexes as they progressed further into puberty (Yuan et al., 2020).

Since both innate and adaptive immunity shapes many of the interactions in the gut microbiome and vice versa, the sexually dimorphic nature of immune systems shows us an association between gut microbiome allergy and autoimmune disorders, as it seems for Intestinal Bowel Disease (Sisk-Hackworth et al., 2023). Currently, it is unclear how immunity and microbiome dimorphism modify the natural history of asthma, but further research may enlighten it.

## 6 Adult, new adaptation

Asthma incidence is lower in adults compared to children, but adult asthma complications have a higher mortality rate (Dharmage et al., 2019). Adults with early-onset current asthma were more prone to atopic conditions and had a higher occurrence of asthma attacks, adult-onset asthma represents a unique phenotype primarily associated with environmental risk factors (Tan et al., 2015). However, He et al., reported through a prospective cohort study that adult-onset asthma has higher all-cause and cardiovascular mortality (He et al., 2021). Determining asthma prevalence in adults is challenging due to reliance on self-reporting and varied approaches in studies. Globally, doctor-diagnosed asthma, clinical/treated asthma, and wheezing prevalence in adults is 4.3, 4.5, and 8.6%, respectively, (To et al., 2012). Recent prevalence ranges from 5.4 to 17.9% depending on the definition and region (Song et al., 2016).

According to the phenotype, Wang et al. aimed to characterize the inflammatory response in acute and stable asthma in adults and children. They found that paucigranulocytic inflammation was the most common phenotype in children and adults with stable asthma. However, in acute asthma, neutrophilic inflammation was more prevalent in adults, while eosinophilic inflammation was more prevalent in children (Wang et al., 2017).

In healthy adults, the most common bacterial phyla in the lungs are *Bacteroidetes*, *Firmicutes*, and *Proteobacteria*. Dominant genera found in bronchoalveolar lavage (BAL) from healthy adults include *Prevotella, Veillonella, Pseudomonas, Fusobacteria*, and *Streptococcus* (Wang et al., 2017; Leviatan et al., 2022). Wang et al., compared the gut microbiota of 185 controls and 36 asthmatic adults in the UK and found *Faecalibacterium prausnitzii, Sutterella wadsworthensis,* and *Bacteroides stercoris* were depleted in cases, while *Clostridium* with *Eggerthella* were over-represented in individuals with asthma (Wang et al., 2018). Concerning the respiratory microbiome in adults with established asthma has been reported an increased abundance of the genera *Streptococcus, Haemophilus, and Moraxella* while a decrease of *Prevotella* and *Corynebacterium*, which is associated with proinflammatory response, associated with severe airway obstruction and airway neutrophilia, through activation of a Th2 response (Hufnagl et al., 2020).

Overall, changes in the microbiome that take place during adulthood can be more reliably linked to health and disease compared to younger individuals. Specific taxa have been found to have associations with both health and disease, indicating the importance of preserving potentially beneficial symbionts (Ghosh et al., 2022).

### 6.1 Environment pollution and microbiome in asthma adulthood

Asthma is a complex condition influenced by several factors, including the immune system, allergens, environmental triggers, and epigenetics. Within this intricate interplay, environmental factors play a significant role. These factors encompass a wide range of biomolecules, such as pollutants, household cleaners, microplastics, nanoparticles, and tobacco smoke.

A study conducted by Gehring et al., provided evidence of a notable increase in asthma occurrence among individuals exposed to pollutants during early life, which can have long-term consequences in adulthood (Gehring et al., 2020). In industrialized countries, where people spend most of their day indoors, the composition of indoor air is affected by various factors, including outdoor pollutants, ventilation quality and quantity, indoor allergens, and activities such as smoking, heating, and cooking. Microorganisms present in indoor air, particularly certain fungal species such as *Aspergillus, Penicillium, and Cladosporium*, have been associated with an increased risk of asthma in both children and adults (Sharpe et al., 2015).

Cigarette smoke contains nicotine, aldehydes, and polycyclic aromatic compounds, which can decrease endogenous antioxidants, increase lipid peroxidation, and induce oxidative stress. Furthermore, these substances can contribute to intestinal dysbiosis. Animal models have shown that cigarette smoking significantly reduces the concentrations of organic acids, such as acetic acid, propionic acid, butyric acid, and valeric acid, as well as the population of *Bifidobacterium* in the cecum, indicating the presence of intestinal dysbiosis (Tomoda et al., 2011). In a study by Pfeiffer et al., *Prevotella, Veillonella, Streptococcus*, and *Actinomyces* were found to be the most abundant genera in the respiratory tract of smokers (Pfeiffer et al., 2021). Furthermore, when evaluating smoking patterns, they observed a negative correlation between the prevalence of *Corynebacterium* and *Dolosigranulum* in nasal samples and the maximum number of cigarettes smoked daily. Simpson et al. examined asthma patients and characterized their sputum microbiota. Their findings showed that ex-smokers had a greater occurrence of the Fusobacteria phylum, as well as higher levels of Firmicutes and Bacteroidetes, while they had lower levels of Proteobacteria when compared to individuals who had never smoked. Additionally, they discovered a connection between smoking and increased bacterial diversity (Simpson et al., 2016). In contrast, Munk et al. report no significant changes in the microbiome of smoking asthmatic patients compared to those who have quit smoking (Munck et al., 2016). Hence, further research is needed to elucidate the connection between smoking and the microbiome in individuals with asthma.

### 6.2 Microbiome and asthma in occupationally exposed workers

Occupational asthma, which accounts for 10 to 25% of asthma cases in adulthood, is the most common form of occupational lung disease. It can be classified into different types based on its etiology, including work-exacerbated asthma, irritant-induced asthma, and immunologic occupational asthma. Low molecular weight isocyanates are particularly prevalent among the compounds responsible for occupational asthma (Kenyon et al., 2012; Maestrelli et al., 2020). Isocyanates have also been linked to dysbiosis observed in other chronic inflammatory diseases such as atopic dermatitis, as they disrupt the symbiotic pathways between *Roseomonas mucosa* and *Staphylococcal* species present on the skin (Zeldin et al., 2023).

Another study conducted by Ahmed et al., focused on ceramics industry workers in a major industrial Egyptian city compared to individuals from a rural village. They found a significant increase in the relative abundance of the Proteobacteria phylum in the industrial group (*p* = 0.02). The industrial group was predominantly populated by *Staphylococcus, Sphingomonas*, and *Moraxella*, leading to the conclusion that environmental pollution may alter the nasal microbiome and disrupt its community structure (Ahmed et al., 2019). While the changes in the microbiota resulting from occupational exposure are well-documented, as are their associations with other inflammatory diseases, establishing a direct link between these changes and the prevalence of asthma remains challenging based on current evidence. However, considering that occupational exposure is part of our life, several occupations could represent a risk factor for the development and exacerbation of asthma through diverse mechanisms.

### 6.3 Viral infections associated with the development of asthma

Respiratory viral infections play a crucial role in the development of asthma and are significant contributors to asthma exacerbations (Hofstra et al., 2015). Among viral infections, HRV infections are particularly common, as this pathogen circulates widely within the community. In adults, viral infections, especially HRV, are responsible for 50–80% of asthma exacerbations, with HRV being detected in up to 83% of adult cases (Jartti et al., 2020; Ojanguren et al., 2022).

In a study involving 88 adults hospitalized for asthma exacerbation, respiratory viruses were detected in 50% of the patients. HRV was the most frequently identified virus (77%), followed by human coronavirus (16%), parainfluenza virus (5%), and human metapneumovirus (2%). Six of these patients also had bacterial coinfections (Bjerregaard et al., 2017). Voraphani et al., reports that individuals who are active smokers and have a history of respiratory syncytial virus infections during the first 3 years are 1.7 times more likely to have current asthma as adults (Voraphani et al., 2014).

Interestingly, in a study of the virome in the sputum of asthma patients, Choi et al. reported an increase in the abundance of Cytomegalovirus (CMV) and Epstein–Barr virus (EBV). Additionally, there was a decrease in Streptococcus phage in patients who experienced exacerbations, which was correlated with more severe disease (Choi et al., 2021).

The precise mechanisms underlying virus-related asthma are still under investigation. However, deficient interferon-γ and interleukin-10 responses, along with an increase in Th2 cytokines such as interleukin-4, interleukin-5, and interleukin-13, have been strongly associated with poor clinical outcomes in the context of viral infections (Busse et al., 2010).

Moreover, respiratory virus infections have been found to induce changes in the composition of the upper respiratory tract microbiota. Infections with rhinovirus, for instance, can predispose individuals to bacterial diseases such as otitis media, sinusitis, and pneumonia (Hofstra et al., 2015). These infections have been linked to an increase in the relative abundance of bacteria such as *Haemophilus, Neisseria, Streptococcus,* and *Moraxella*. These alterations in bacterial composition have been associated with a neutrophilic airway phenotype and persistent asthma that is resistant to treatment (Ver Heul et al., 2019).

That is why it is necessary to continue investigating the complex interplay between environmental exposures, viral infections, dysbiosis, and asthma. Through this exploration we can gain valuable insights into the mechanisms driving asthma exacerbations and potentially develop new strategies for prevention and treatment.

## 7 Elderly, everything has changed

Aging refers to all natural and progressive physiological changes that lead to cellular senescence and a gradual decline in the organism’s biological functions and metabolic stress adaptability. Certain biomarkers help determine aging, such as bone density, frailty, muscle mass, cognitive function, cardiovascular health, some blood biometrics and chemistry parameters, and telomere length (López-Otín et al., 2013), and the microbiome (Partridge et al., 2018). For this reason, chronic systemic inflammation, immunosenescence, and microbiome changes are important for understanding aging diseases such as asthma.

### 7.1 Metabolic changes and comorbidities

Comorbidities are a fundamental factor when studying asthma. They can occur at all ages, however, from adulthood onwards they become more important. Yañez et al., showed that of a total of 152 elderly people with asthma, 36% had three or more comorbidities (Yáñez et al., 2018). Obesity, metabolic syndrome (MetS), and diabetes are among the most common metabolic disorders related to asthma, due to their high prevalence (Park et al., 2018). Furthermore, adipose tissue mass is positively related to high levels of proinflammatory molecules like leptin, IL-6, and TNF-a, and negatively related to anti-inflammatory markers such as adiponectin. Similarly, inflamed adipose tissue releases adipokines that circulate to the lungs and contribute to hyperresponsiveness (Shore, 2010; Park et al., 2018; Palma et al., 2022).

A connection has been established between dysbiosis and obesity, characterized by a decrease in the diversity of bacterial genera that constitute the microbiota (Kim et al., 2021). A study by Fu et al. reported that the richness of bacterial microbiota correlates negatively with body mass index and serum triglyceride levels, while positively correlating with serum High-density lipoprotein levels (Fu et al., 2015). Likewise, it has been reported that individuals with obesity and severe asthma showed an increase in taxa belonging to family Prevotellaceae, Mycoplasmataceae, Lachnospiraceae, and Spirochaetaceae (Huang et al., 2015).

The relationship between intestinal microbiota alterations and the improvement of asthma symptoms remains poorly understood. However, it is speculated that increased production of SCFAs, particularly butyrate and propionate, reduces pro-inflammatory cytokines and/or increased immunoregulatory cytokines (Kim et al., 2021). Nevertheless, these associations have been primarily described within the context of the intestinal microbiota, highlighting the need for further investigation of the relationship between asthma, obesity, and pulmonary microbiota dysbiosis.

### 7.2 Microbial succession in the final stage

Late succession has become very important as a subject of study because of the direct relationship between it and healthy aging. Over time, the alpha diversity of the microbiota decreases, and beta diversity increases, making older adults more susceptible to infection by opportunistic bacteria (Martino et al., 2022). In 2016, Biagi et al. showed that the gut microbiota of elderly people is dominated by the families Bacteroidaceae, Lachnospiraceae, and Ruminococcaceae (Biagi et al., 2016). However, other families such as Prevotellaceae, Enterococcaceae, Lactobacillaceae, Turicibacteraceae, Christensenellaceae, Clostridiaceae, Enterobacteriaceae, Bifidobacteriaceae, Porphyromonadaceae, Peptostreptococcaceae, and Coriobacteriaceae were also found. It has been described that the elderly generally has a gut microbiota composed predominantly of Firmicutes, Tenericutes, Actinobacteria, Lachnospira, and Proteobacteria.

Nevertheless, a decline in taxa such as *Prevotella, Eubacterium, Bifidobacterium, Faecalibacterium, Coprococcus*, and *Roseburia* is observed with increasing age. In contrast, *Akkermansia, Odoribacter, Butyricimonas, Butyrivibrio, Oscillospira, Christensenellaceae*, and *Barnesiellaceae* have been found to increase in abundance in older adults, which has been associated with healthy aging (Biagi et al., 2010; Claesson et al., 2012; Park et al., 2015; Biagi et al., 2016; Effendi et al., 2022). The gradual decline of some microorganisms directly affects systemic inflammation and disease development in the elderly. Additionally, some conformational changes are also associated with unhealthy aging, such as an increase in pathogenic microorganisms like *Eggerthella, Bacteroides, Desulfovibrio, Enterobacteriaceae, Campylobacter, Streptococcus, Actinomyces,* and *Clostridium species.*

Even though the gut microbiome is the best studied, organisms in the respiratory tract are also important when studying respiratory diseases such as asthma. A study reported by Lee et al., examined the composition of airway microbiota in young adults and elderly individuals, comparing those with and without asthma. The dominant phyla in young adults and elderly groups were Actinobacteria, Firmicutes, Proteobacteria, and Bacteroidetes, but their relative abundances differed significantly. Additionally, the research noted a higher prevalence of Moraxella in elderly individuals without asthma compared to their asthmatic counterparts (Lee et al., 2019). Recently, centenarian gut microbiota have been found to undergo new compositional changes despite differences or similarities between different populations. This has sparked interest in their study, as an increase in the abundance of genera such as *Akkermansia*, *Bifidobacterium, Christensenellaceae*, and other species associated with healthy aging has been described (Biagi et al., 2016; Kato et al., 2017). However, further studies need to be conducted to understand these relatively recent findings.

### 7.3 Immunosenescence and chronic inflammation

Immunosenescence is a multifactorial phenomenon in which both innate and acquired immunity are affected over time, impairing the effective immune response against pathogens, pathobionts and antigens (Van Den Munckhof et al., 2020). It is thought to result from a combination of three factors: Autoimmunity, Immunodeficiency, and Immune dysregulation (Chotirmall and Burke, 2015). The increase in pro-inflammatory cells leads to a chronic low-grade inflammatory state known as “inflammaging.” This inflammaging is a synergistic process between immunosenescence, chronic disease, and the microbiome in which older adults become vulnerable to potentially dangerous bacteria and increase the risk for diseases such as diabetes, obesity, heart disease, and asthma (Chotirmall and Burke, 2015; Huang et al., 2020).

Chronic inflammation is also mediated by the abundance of certain microorganisms. Short-chain fat-producing genera such as *Faecalibacterium, Roseburia, Lachnospira, Eubacterium, Coprococcus, Butyricimonas*, and *Butyrivibrio* have been studied to maintain immune homeostasis by downregulating proinflammatory mediators (Serrano-Villar et al., 2017; Effendi et al., 2022; Singh et al., 2023). Unfortunately, the progressive decrease of these genera leads to a deficiency of SCFA, which increases the permeability of the intestinal mucosa. For this reason, genera such as *Akkermansia* become more important as they are acetate producers (Bodogai et al., 2018; Wu et al., 2021). In contrast to the above genera, the increase of *Bacteroides* is associated with low-grade inflammation, as shown in a study conducted in Korea by Lim et al. (2021). This study also showed that increases in *C. hathewayi* positively correlate with activation of proinflammatory Th17 cells. Similarly, some *Campylobacter* strains produce cytolysin toxins that induce hyperinflammatory proteins, and *Desulfovibrio* oxidizes butyrate (Callahan et al., 2021).

Some microorganisms help regulate the immune system. *Enterococcus faecalis* is a ROS-producing species involved in oxidative metabolism. However, its progressive increase contributes to inflammation, increased apoptosis, and contributes to oxidative damage to mitochondrial and nuclear DNA (Hemachandra Reddy, 2011; Bullone and Lavoie, 2017). *Bacteroides fragilis* produces PSA, a polysaccharide that binds to B cells inducing CD4+ and CD8+ regulatory T cells, thereby secreting IL-10 (Ramakrishna et al., 2019). It has also been suggested that this species may stimulate and differentiate Treg cells and thus participate in immune regulation (Troy and Kasper, 2010; Johnson et al., 2015; Wu et al., 2021). A study by Li et al. (2023), showed that species such as *Lactobacillus fermentum* and *Bacteroides fragilis* play a role as probiotic strains. Their combined use in senescent mice improved neuronal cell necrosis, antioxidant capacity, and reduced inflammation levels (Li et al., 2023).

### 7.4 Microbiome and asthma in elderly people

Asthma is a heterogeneous phenotypic disease that has not been fully characterized in the elderly (Liu et al., 2020). However, it is known that reversible obstruction, hyperresponsiveness, and chronic airway inflammation are representative features of this disease. The deterioration of the immune system, systemic inflammation, impaired lung function, different phenotypes, airway remodeling, comorbidities and late onset of this disease complicate its investigation and treatment (Zhang and Huang, 2021). Allergens, tobacco, pollution, and diet are also directly involved in the development of the disease. With age, microbiome alterations in the elderly lead to opportunistic microorganisms colonizing the lungs as environmental conditions are optimal for their development and the immune system is less effective in eliminating them (Santacroce et al., 2020).

A study by Lee et al. analyzed the composition and functional profile of the microbiota in asthmatic and non-asthmatic elderly in Seoul (Lee et al., 2019). The genera *Burkholderia* and *Psychrobacter* were positively correlated with lower forced expiratory volume (FEV1). Therefore, the authors suggested that the low abundance of these microorganisms might be related to asthmatic features. It was also suggested that the increased abundance of *Corynebacterium* might be related to the development of asthma, as this genus has been described in other respiratory diseases such as rhinosinusitis.

Although asthma directly affects the airways, it has been discovered that the gut microbiota can be associated with lung function and asthma. Begley et al., showed that the gut microbiota of older adults in Michigan is certainly dominated by some Prevotella species and that they are associated with chronic inflammatory diseases (Begley et al., 2018). *Staphylococcus aureus* is a microorganism of the microbiota involved in the pathophysiology of airway diseases, including asthma. A study by Song et al., showed that staphylococcal enterotoxin IgE (SE-IgE) is significantly associated with asthma in the elderly, particularly with late-onset asthma (Song et al., 2016). This species has also been shown to produce staphylococcal enterotoxin B (SEB) which can induce Th2 polarization, inflammation and corticosteroid resistance and can inhibit regulatory T-cell functions in humans (Hauk et al., 2000; Cardona et al., 2006).

The elderly are a highly vulnerable sector of the population, diminished by the effects of aging and various diseases. For this reason, the study of the microbiota in the elderly has become a useful and fundamental tool to understand pathologies, find new treatments, and create an adequate culture of prevention. Research on the microbiota and its relationship with diseases such as asthma has been limited; however, understanding this relationship may lead to useful insights for people of other ages.

## 8 Effects of asthma treatment on the composition of microbial diversity

International Asthma guidelines define corticosteroids as the key asthma treatment (Reddel et al., 2022). These anti-inflammatory molecules inhibit the recruitment of immune cells in the airway by suppressing the production of IL-1B, IL-6, GM-CSF, ICAM-1, induce eosinophils apoptosis, and diminish the survival of T-lymphocytes and mast cells (Barnes, 2010). Usually inhaled corticosteroids (ICS) are enough to control symptoms and reduce complications, but during asthma exacerbations, higher doses are needed and sometimes Oral Corticosteroids (OCS) therapy is required, with increased adverse reactions because of their systemic effects (Perez-Garcia et al., 2020).

Zhou et al., conducted a longitudinal study to identify changes in nasal microbiota related to the risk of asthma exacerbations despite ICS therapy (Zhou et al., 2019). Nasal swabs were collected among 214 European children with mild–moderate asthma, at the time of well-controlled and during the first loss-asthma-control episode. Patients with nasal microbiome dominated by *Corynebacterium* and *Dolosigranulum* had fewer episodes of exacerbation and longer time between them compared to those with predominant *Staphylococcus, Streptococcus*, or *Moraxella* genera. Bacterial richness increased during exacerbations compared with well-controlled asthma. Furthermore, a higher relative abundance of *Corynebacterium* was associated with a lower risk of asthma exacerbations requiring OCS use, whereas *Moraxella* was associated with a higher risk of requiring OCS (Zhou et al., 2019).

Immune modulatory effects of corticosteroids might change the respiratory microbiome. Huang et al. evaluate the effect of ICS on microbiome composition, they studied asthma patients over 9 months, sampling before dosage, three months later, and nine months after the start of treatment. Genera *Streptococcus, Rothia, Actinomyces, Leptotrichia,* and *Neisseria* were identified as the predominant in all samples without significant differences between them or in alpha diversity during the study. More than two-fold decrease the percentage of Wallemia, Cladosporium, Penicillium and Alternia genera compared with baseline, concurrently with decrease in bacteria-fungus intra and inter-kingdom networks after ICS therapy (Huang et al., 2022). Martin et al., compared low- and high-dose ICS groups in sputum microbiome composition without significant differences in bacterial load or overall community (Martin et al., 2020). However, Streptococcus genera showed significantly higher relative abundance in subjects taking low-dose ICS and *Haemophilus parainfluenzae* was significantly more abundant in subjects on high-dose fluticasone propionate than those on high-dose budesonide over a 2-week period. Denner et al., studied the bronchial microbiome and correlated OCS use with a decrease in the relative abundance of Bacteroidetes and Fusobacteria with an increase in Proteobacteria phyla, generalized linear models on brush samples demonstrated OCS usage influence the relative abundance of *Pseudomonas, Rickettsia, Lactobacillus* and *Streptococcus* genera, significantly enriched in asthmatic patients sample (Denner et al., 2016). In addition, α-diversity in brush samples from asthmatic subjects was correlated with lowest FEV1 levels, a clinical parameter of airway obstruction.

Goleva et al., showed that asthma patients resistant to corticosteroid treatment occur due to the expansion of specific gram-negative bacteria in the airways, like *Haemophilus parainfluenzae*, the LPS of item interact with Toll-like receptor 4 and activate transforming growth factor-b-associated kinase-1 (TAK1), by MyD88 pathway resulting in the p38 MAPK activation and Nuclear Factor-kappa Beta (NF-kb), increasing the production of proinflammatory cytokines like IL-8, also activation of TAK1 inhibits the production of MKP-1 mediated by glucocorticoid receptor, this results in reduced cellular responses to corticosteroids and reduction of sensitivity to them (Goleva et al., 2013).

These studies show the complex correlation between microbiome and corticosteroids, enlightening the need for more research to better understand the phenomena and its implications for better and more reasonable treatment of asthma patients.

## 9 New perspective for asthma treatment: probiotics

The adaptive immune system provides versatile defense against infectious agents but faces the challenge of potential autoimmune inflammation due to T cell self-reactivity. Tregs play an important role in preventing autoimmunity. Tregs can reverse fatal autoimmunity, tissue pathology, and offer long-term protection (Hu et al., 2021).

Probiotics have the potential to modulate various types of immune cells, including T helper (Th)-1, Th2, Th17, Treg cells, and B cells, which play an important role in human health and the development of immune-related disorders. The use of probiotics has been associated with the modulation of the severity of allergic inflammation (Dargahi et al., 2019). The beneficial effect induced by probiotics is based on their ability to act as an “on/off” switch to control immune responses in a strain-dependent manner at the mucosal level. In allergic asthma, they protect the immune system’s homeostasis by regulating the balance between Th1 and Th2 cells, reducing the inflammatory response, modulating the gut microbiota, and increasing the number of Tregs (Huang et al., 2021).

In a study conducted by Wu et al., a probiotic formulation comprising *Lactobacillus acidophilus, Lactobacillus rhamnosus,* and *Bifidobacterium animalis* demonstrated the ability to regulate peribronchial inflammation and control the expression of the PI3K gene in individuals with allergic asthma (Wu et al., 2022). A systematic review conducted by Lin et al., revealed that supplementation with probiotics may reduce the number of asthma episodes (Lin et al., 2018). However, no significant improvements were observed in terms of symptoms during the daytime or nighttime, as well as pulmonary function measures such as FEV1 and PEF. Nevertheless, the authors emphasize the importance of further well-designed randomized controlled trials with larger sample sizes to fully evaluate the effects of probiotics in children with asthma.

## 10 Conclusion

The interplay between the host microbiome and asthma exhibits significant variations across diverse contexts, and whether a microbiome is considered healthy or disease-associated depends on the context (Table 1). A comprehensive understanding of these intricate interactions among variables and the microbiome is essential for unveiling the underlying mechanisms of asthma phenotypes and for developing precise interventions for prevention and treatment.

**Table 1:** Microbiota profiling in asthmatic and healthy individuals.

Sample method	Population	Asthmatic subjects	Healthy controls (Ref)
*Superior airway*			
Nasopharyngeal aspirate^S^	*Infants* (0–12 months) *n* = 234	Increased *Streptococcus.* Strong asthma predictor. *Streptococcus, Moraxella, or Haemophilus* marked virus-associated with acute respiratory infections in asthma.	Dominated by *Staphylococcus* (41%) and *Corynebacterium* (22%). Antibiotic usage in the four weeks prior to sampling was associated with higher abundances of *Haemophilus*, *Streptococcus*, and *Moraxella* and lower abundances of *Alloiococcus* and *Corynebacterium* (Teo et al., 2015).
Nasal swab^S^	*Children and adolescents* (6–20 years) *n* = 14	Increased *Moraxella Catarrhalis, Escherichia* and *Psychrobacter*. Dominated by *M. Catarrahalis* and less diverse.	*M. catarrhalis* less abundant *Corynebacterium tuberculostearicum*. Found in high abundance (Castro-Nallar et al., 2015).
Nasal swab^S^	*Adults* (35.8 +/− 16) *n* = 72	Increased *Bacteroidetes (Prevotella), Proteobacteria (Alkanindiges), Actinobacteria (Gardnerella).*	Less *Proteobacteria and Bacteroidetes* (Fazlollahi et al., 2018).
Nasopharyngeal swab^S^	*Elderly* (<60 years) *n* = 40	Higher relative abundance of *Moraxella*. Higher abundance of *Proteobacteria.*	Higher relative abundance of *Corynebacteriales* (Lee et al., 2019).
Oropharyngeal swab^S^	*Elderly* (53.4+/− 17.1//55 +/− 13) *n* = 47	*Proteobacteria* (*Pseudomonas* s) and *Firmicutes* (*Lactobacillus spp*) are the most dominant populations in asthmatic subjects, these microorganisms not detected in healthy subjects.	*Proteobacteria* (*Pseudomonas*) and *Firmicutes (Lactobacillus* spp) no detected in healthy subjects *Bacteroidetes* (*Streptococcus, Veillonella, Prevotella, and Neisseria*) dominant in healthy oropharynx (Park et al., 2014).
Hypopharyngeal aspirate ^C^	*Neonates* (~1 month) *n* = 321	Neonates colonized with *Streptococcus pneumoniae, M. catarrhalis, Haemophilus influenzae* showed increased asthma prevalence at 5 years.	Neonates not colonized with *S. pneumoniae, M. catarrhalis or H. influenzae* show less risk of a first wheezy episode (Bisgaard et al., 2007).
Hypopharyngeal aspirate^S^	*Children* (12–36 months) *n* = 68	Increase abundance of *Moraxella, Haemophilus* and *Streptococcus*, being *Moraxella* the predominant genera with mean relative abundance of 43.63%.	No healthy control was included (Thorsen et al., 2021).
Broncho-alveolar lavage (BAL)^S^	*Children* (11.8 +/− 2.8 years) *n* = 20	Increase *Proteobacteria* (*Haemophilus*) *and Firmicutes* (*Streptococcus*) in asthmatic children.	Increase *Bacteroidetes (Prevotella)* in healthy subjects (Hilty et al., 2010).
*Inferior airway*			
Broncho-alveolar lavage (BAL)^S^	*Children* (11.8 +/− 2.8 years) *n* = 20	Increase *Proteobacteria (Haemophilus) and Firmicutes (Streptococcus)* in asthmatic children.	Increase *Bacteroidetes (Prevotella)* in healthy subjects (Hilty et al., 2010).
Sputum^S^	*Adults* (39–62 years) *n* = 97	Main species present in airway of healthy and asthmatics patients include *Streptococcus Mitis, Streptococcus Aliviarus* and *Veillonella Dispar.*	Airway microbiota similar to asthmatic patients. No differences in airway diversity between asthmatic patients and healthy controls in the composition of microbiota (Ham et al., 2021).
Bronchial brushing ^S^	*Adults* (20–63 years) *n* = 40	Increased *Bacteroidetes* and *Firmicutes* in severe asthma. Increased *Actinobacteria (Mycobacteria, Streptomyces)* and *proteobacteria (Klebsiella).*	Less abundant in *Proteobacteria (Klebsiella)* (Huang et al., 2015).
Induced sputum^S^	*Adults* (age 56–59) *n* = 167	Significant decrease of alpha diversity in neutrophilic phenotypes High abundance of *Moraxella* and *Haemophilus* in neutrophilic phenotypes. Negative correlation of sputum neutrophil percentages with *Gemella*, *Porphyromonas* and *Streptococcus* Taxa.	No healthy control was included (Taylor et al., 2018).
*Gut*			
Broncho-alveolar lavage (BAL)^S^	*Children* 11.8 +/− 2.8 years) *n* = 20	Increase *Proteobacteria (Haemophilus) and Firmicutes (Streptococcus)* in asthmatic children.	Increase *Bacteroidetes (Prevotella)* in healthy subjects (Hilty et al., 2010).
Fresh stool^S^	*Adults* (18–50 years) *n* = 67	Lower alpha diversity enrichment of *Ruminococcus gnavus, Clostridium clostridioforme,* and *Bifidobacterium pseudocatenulatum* Depletion of *Roseburia intestinalis* and *Roseburia inulinivorans.*	Richer alpha diversity Enrichment of *Roseburia inulinivorans* and *Clostridium disporicum* (Zou et al., 2021).
Fecal stool^S^	*Adults* (39–62 years) *n* = 97	At genus level, the leading bacteria are *Prevotella, Bacteroides, Faecalibacterium,* and *Rominococcus*. The most common species were *Prevotella Copri, Faecalibacterium prausnitzii*, and *Bacteroides Plebeius.*	Similar to asthmatic patients. There were no significant differences between groups or associations between gut microbiota composition and asthma (Ham et al., 2021).

This table shows the microbial diversity in patients with asthma compared to other study groups, considering different life stages and respiratory/gut tract locations. Analysis Method. S: 16S rRNA gene sequencing. C: Culture. Ref.: Reference.

The microbiome has been shown to play a significant role in early-life immune development and modulation. It’s crucial to note that this interaction can be influenced by genetic factors, the mode of birth (vaginal delivery or cesarean section), first feeding method (breast or formula), upbringing environment (rural or urban, presence of older siblings or pets), among others. In early-life, it has been reported that certain genus in the gut microbiome, including *Bifidobacterium, Lactobacillus, Faecalibacterium*, and *Bacteroides*, play a protective role against asthma. During adolescence, changes in the microbiome occur, partially influenced by the maturation of the immune system and hormonal changes during this stage. As individuals reach adulthood, the impact of the microbiome on health and disease becomes more apparent, contributing to the development of chronic conditions that can lead to comorbidities and proinflammatory states that predispose individuals to asthma and other diseases in old age. In adults, there is evidence indicating an increase in the presence of *Clostridium* and *Eggerthella* in the gut microbiome of individuals with asthma. Moreover, genus such *as Haemophilus, Streptococcus, Staphylococcus*, and *Moraxella* in the respiratory microbiome have emerged as significant contributors to the pathogenesis of asthma. Additionally, the reduction of the genus *Corynebacterium* in the respiratory tract during early-life and adult stages has been associated with proinflammatory responses in specific contexts. However, this genus may also be linked to the development of asthma, particularly among the elderly population.

To illustrate this concept, let us consider a forest with its plant and animal species. The population and composition of these organisms can vary significantly based on whether the forest is in a temperate, equatorial, or Mediterranean climate. Moreover, the roles these organisms play are influenced by seasonal changes, creating distinct contexts. Much like this ecological example, the microbiome’s impact on various stages of human growth and development also demonstrates dynamic variations, including its relevance to asthma.

Our review has some limitations; many studies are confined to cohort designs, lacking long-term longitudinal data on individual changes. Furthermore, obtaining samples from the lower respiratory tract to investigate this microbiome remains challenging, and much of the information is derived from the gut microbiome. Moreover, a substantial portion of these studies relies on the sequencing and analysis of the 16S ribosomal gene, limiting the scope to bacterial aspects and gender-based analyses. Consequently, a comprehensive understanding of the virome and mycobiome is still needed. Therefore, further research and long-term follow-up studies are necessary to fully elucidate the mechanisms underlying these interactions and explore potential interventions, such as probiotics, that can modulate immune responses and improve health outcomes in diverse populations worldwide.

## Author contributions

DG-C: Conceptualization, Data curation, Formal analysis, Investigation, Methodology, Project administration, Validation, Visualization, Writing – original draft, Writing – review & editing. IG-G: Formal analysis, Investigation, Writing – original draft, Writing – review & editing. KL-S: Formal analysis, Investigation, Writing – original draft, Writing – review & editing. VI-M: Formal analysis, Investigation, Writing – original draft, Writing – review & editing. FJ-J: Formal analysis, Investigation, Writing – original draft, Writing – review & editing. AT-G: Data curation, Formal analysis, Investigation, Writing – original draft, Writing – review & editing. CB-C: Formal analysis, Investigation, Validation, Writing – original draft, Writing – review & editing. MM-M: Formal analysis, Investigation, Visualization, Writing – original draft, Writing – review & editing. LJ-A: Formal analysis, Investigation, Visualization, Writing – original draft, Writing – review & editing. JZ: Conceptualization, Formal analysis, Funding acquisition, Investigation, Project administration, Supervision, Visualization, Writing – original draft, Writing – review & editing. AC: Conceptualization, Data curation, Formal analysis, Funding acquisition, Investigation, Project administration, Resources, Supervision, Validation, Visualization, Writing – original draft, Writing – review & editing.
